# Supplementary material for: Complexome profiling of the Chlamydomonas psb28 mutant reveals TEF5 as an early PSII assembly factor
Source: Plant Cell. 2025 Mar 17;37(6):koaf055. doi: 10.1093/plcell/koaf055 (PMC12164590; doi:10.1093/plcell/koaf055)
Supplement: koaf055_Supplementary_Data [file koaf055_supplementary_data.zip › psb28_interactive.zip!/plots/intensity/qualityPlots/peptides/HSLU1_Cre12.g510900.t1.2.html]

### Cre12.g510900.t1.2

Trivial name: HSLU1  
SeqCoverage [median]: 10.20  
Mass: 66.45 kDa  
  
 

| Sequence | MissedCleavage | Mass [Da] | wtIntensity | muIntensity | Score |
| --- | --- | --- | --- | --- | --- |
| AVAVAYR | 0 | 748.42 | 360.20 | 0.00 | 69.39 |
| ILTEPEYNLIK | 0 | 1331.73 | 0.00 | 1030.75 | 57.60 |
| QDHLGGADALR | 0 | 1151.57 | 384.06 | 0.00 | 51.45 |
| SGDPSSEGVQR | 0 | 1117.50 | 278.74 | 0.00 | 93.11 |
| TLDNIGAR | 0 | 858.46 | 336.46 | 448.28 | 65.25 |
| VAEEVNR | 0 | 815.41 | 519.08 | 1338.74 | 122.79 |
|  |  |  | 1878.5 | 2817.8 |  |
